# Fatal child abuse in Japan: does a trend exist toward tougher sentencing?

**DOI:** 10.5249/jivr.v3i2.73

**Published:** 2011-07

**Authors:** Saori Nambu, Ayako Nasu, Shigeru Nishimura, Akiyoshi Nishimura, Satoshi Fujiwara

**Affiliations:** ^*a*^Department of Forensic Medicine, Yokohama City University, Graduate School of Medicine, Yokohama, Japan.; ^*b*^Department of Forensic Medicine, Institute of Health Biosciences, The University of Tokushima Graduate School, Japan.; ^*c*^Department of Forensic Medicine, Yokohama City University, School of Medicine, Japan.

## Abstract

**Background:**

It has been pointed out in Japan that criminal punishment in domestic homicide cases, especially in fatal child abuse cases, tends to be more lenient than in public homicide cases that occur outside the home. In recent news accounts of fatal child abuse cases, however, the media has reported that court-imposed sentences have tended to be stricter every year.

**Methods:**

Using the online databases of three major Japanese newspapers, we collected articles about fatal child abuse cases that had been published from January 2008 to December 2009. We analyzed these articles to determine, whether a tendency towards tougher penalties, as was put forward by the media, actually exists at present time in the criminal system in Japan.

**Results:**

We found 24 cases, out of which 20 involved only one offender and 4 involved two offenders. These 28 offenders comprised nine biological fathers, 11 biological mothers, and eight other male relatives of the child victims. We found that the sentences handed down by the court clearly tended to be more lenient for female offenders.

A new system of criminal jurisprudence, the so-called saiban-in system wherein citizens serve as "lay judges" in criminal trials involving serious crimes, was implemented in Japan at the start of 2009. Each, district court has gradually adopted this new system after a preparation period of approximately five years starting in 2004.

**Conclusions:**

Many figures in the Japanese media predicted that the gap between social expectations and court sentences for sanction against domestic homicide cases would be filled with the present transitional period of the Japanese criminal system. However, the present study found no significant difference in the laws regarding sentencing in fatal child abuse cases before and after the preparation period of the saiban-in system.

## Introduction

Recently there has been a rapid increase in the number of child abuse cases in Japan.^1^The number of these cases investigated by the police reached 300 in 2007. According to the National Police Agency (Japan), mothers were the most common abusers, (being implicated in 97 cases) followed by fathers, adoptive fathers, and common-law husbands who were named in 91, 55, and 46 cases, respectively.^2^Yasumi and Kageyama^3^identified 309 fatal child abuse cases in the Japanese newspaper database for the 12-year period from 1994 to 2005, and they found that mothers and fathers were equally responsible for filicide (40.3% were mothers, 39.6% were fathers, and 20.1% of the cases involved both parents). Although they did not mention the offender’s gender, they identified 303 oyako-shinju (filicide-suicide) cases in the same research. Earlier research conducted by Takahashi^4^showed that mothers were overwhelmingly the majority of oyako-shinju offenders in Japan. According to the Japanese Ministry of Justice, a total of 615 murder cases (including in public and domestic) were reported in 2006. Of this number, 521 cases (85%) were committed by males.^5^Therefore; this remarkably high participation rate of women in domestic homicide cases was noteworthy.

It has been pointed out that sentences handed down by Japanese criminal courts in domestic filicide cases tend to be more lenient than those given for public homicide cases.^6^We have assumed that these relatively light sentences for domestic homicide were due to the fact that women belonged to the largest group of offenders. After conducting research on sex differences in sentencing practices of cases in domestic homicide,^7^we found that the sentences given for domestic homicide cases (and also for those of fatal child abuse) were more lenient than those given in murder cases that had occurred outside the home. Moreover, in domestic cases, female offenders were treated more leniently than male offenders, who were convicted almost in the same level as in public homicide cases.

It should be noted that a new criminal procedure, the so-called saiban-in system^8^wherein citizens serve as lay judges in criminal trials involving serious crimes, was implemented in Japan beginning on May 21, 2009. Under the new system, in contrast to the traditional criminal court where a professional judge(s) makes a decision exclusively, six eligible voters work with three professional judges to decide on the verdict and the appropriate sentence. Each district court has gradually adopted this new system, after a preparation period of approximately 5 years since 2004. Measures to adapt to the new system have gained momentum across the country since 2008. The Japanese news media has occasionally pointed out that an expectation gap exists between strict and lenient court sentences for defendants in domestic homicide cases. The “Heki-chan case,” which occurred in 2006, exemplifies such a gap.

The father (25 years old) and mother (28 years old) kept on beating their son Heki (3 years old) for hours with a metallic mop because he was getting on their nerves. They put him into a bathtub full of cold water (10℃), made him sit down with his buttocks on top of his ankles (a Japanese-style common courtesy called Seiza), and they left him unattended for two hours without any intervention. Post-traumatic shock (Reducing the volume of circulating blood due to multiple contusions over his entire body) was considered to be the cause of his death. In this case, the prosecutor demanded a 13-year prison term for the father and a 12-year term for the mother, which is an unusually severe penalty in Japan for “Bodily Injury Resulting in Death.”^9^The court did find both parents guilty but the Kyukei was too severe, and sentenced the father to 7 years and the mother to 6.5 years in jail.^10^

With regard to differences in the process of sentencing on the basis of the offender’s sex, we researched whether, as the media had reported, the trend towards tougher penalties under the present transitional period of criminal system in Japan existed. 

## Methods

Using online databases of three major newspapers (Asahi Shinbun, Yomiuri Shinbun and MainichiSshinbun), we collected articles using the search terms ‘gyakutai’ (abuse), ‘chishi’ (fatal), ‘kyukei’ (procecutor-suggested sentences), and ‘hanketsu’ (court sentence) for a period of 24 months from January 2008 to December 2009. Later we reviewed the entire text of articles containing selected cases in which offenders had been charged with a single count of Bodily Injury Causing Death against a sole child (below the age of 15 years). The kyukei (which is a unique feature of the Japanese criminal jurisprudence), the court sentence, and the ratio of the court sentence to the kyukei (hereafter referred to as “ratio”) in each case were identified. Newspaper articles on the same case appearing in several newspapers were entered once, although in order to create a full profile for each case, we reviewed the entire text of each article and abstracted the following information: (1) Offender’s gender/ age, (2) Victim’s gender/ age, (3) relationship between Offender and victim, (4) the cause of Victim’s death.   When additional details were required, we conducted keyword searches in an Internet search engine (Yahoo! Japan) using the offender’s name and chose the most reliable information as provided by official organization or the press. Data were analyzed using Microsoft Excel 2007.

## Results

We found 24 cases, out of which 20 involved only one offender  and  4  involved  two  offenders.  These   28   offenders comprised nine biological fathers, 11 biological mothers, and eight other male relatives of the child victims.Table 1-a and b  represent the case details for male and female offenders.Table 2represents couple offenders. Of all single offenders, thirteen were male and seven were female. Fifteen of their biological children were their victims. Of the 24 victims, sixteen were boys and the other eight were girls. However, of the thirteen victims abused by male offenders, eleven were boys (84.6%), whereas in contrast, of the seven victims abused by females, five were girls (71.4%), indicating gender of the victim and the perpetrator were correlated.

**Table:** Table 1-a:**Case Details of Male Offenders**

Case(age)	Victim sex/age	Relationship	Offence	Cause of death	Kyukei(years)	Sentence(years)	ratio
Ｍ1(27)	F/ 2y	Biological	punched and kicked her face and neck	Head injury	8	6	0.75
Ｍ2(32)	M/ 11y	Non- biological	lifted him up and threw him onto the bed	Head injury	8	7	0.88
Ｍ3(22)	F/ 6y	Non- biological	beat her face and neck, strangulated and shook her	Head injury	10	7	0.7
Ｍ4(31)	M/ 5y	Non- biological	impacted his head twice against the floor	Head injury	10	7	0.7
Ｍ5(34)	M/ 1y	Biological	tossed him over, suffocated with a quilt	Suffocation	10	7	0.7
Ｍ6(24)	M/ 2m	Biological	lifted him up and threw him onto the floor	Head injury	8	6	0.75
Ｍ7(24)	M/ 7m	Biological	beat him in the face several times	Head injury	8	5	0.63
Ｍ8(29)	M/ 3m	Biological	beat him in the stomach	Intra-abdominal bleeding	8	7	0.88
Ｍ9(22)	M/ 3y	Non- biological	lifted up and suffocated with a quilt	Head injury	8	6	0.75
Ｍ10(31)	M/ 1y	Biological	beat his face and chest, upset and hit his head against the floor and a post	Head injury	6	4.5	0.75
Ｍ11(39)	M/ 3y	Non- biological	beat and hit his head against a table and a chair	Head injury	7	4	0.57
Ｍ12(23)	M/ 5m	Biological	shook his head violently	Head injury	7	5	0.71
Ｍ13(37)	M/ 9m	Biological	knocked him down, laid him on the stomach and bent him over backward to put his toes to the occipital region by force or stepped on the stomach	Reducing the volume of circulating blood due to the fractures	10	9	0.9

“M”=Male, “F”=Female, “y”= year old,” m”=month old,  “ratio”= ratio of the court sentence against the Kyukei

**Table:** Table 1-b:**Case Details of Female Offenders**

Case(age)	Victim sex/age	Relationship	Offence	Cause of death	Kyukei(years)	Sentence(years)	ratio
Ｆ1(23)	F/ 3y	Biological	beat and knocked against her head	Head injury	7	4.5	0.64
Ｆ2(21)	M/ 1y9m	Biological	impacted against a floor after shaking	Head injury	5	3	0.6
Ｆ3(29)	F/ 3y	Biological	hit, knocked down and knocked head on the floor	Head injury	8	4	0.5
Ｆ4(24)	M/ 2y	Biological	pushed and stepped on stomach	Pancreaticrhexis	8	5	0.63
Ｆ5(28)	F/ 4m	Biological	impacted head against a quilt	Head injury	5	Suspend (0)	0
Ｆ6(32)	F/ 5y	Biological	shook violently or impacted against a quilt	Head injury	10	5	0.5
Ｆ7(23)	F/ 1y	Biological	tramped on the stomach	Hepatorrhexis+intra-abdominal bleeding	8	4	0.5

“M”=Male, “F”=Female, “y”= year old,” m”=month old, “ratio”= ratio of the court sentence against the Kyukei

For male offenders, the kyukei ranged from 6 to 10 years (8.3 ± 1.26 years), court sentence ranged from 4 to 9 years (6.2 ± 1.29 years), and ratio was 0.74 ± 0.07. For female offenders, the kyukei ranged from 5 to 10 years (7.3 ± 1.67 years), the court sentence ranged from 0 (suspended sentence); When she receives suspended sentences of imprisonment, she is immediately released from custody. When a further crime is committed within the period of suspension and imprisonment is imposed for the crime, however, suspension of the execution of the sentence shall be revoked (JPC Article 26). In Japanese society, the treatment regarded as discharge substantially; to 5 years (3.6 ± 1.61 years), and the average ratio was 0.56 ± 0.07.

There were four cases in which both males and females were found to have jointly committed the offence of the Bodily Injury Resulting in Death (Table 2) . In three of the four cases, the kyukei and the court sentences for both male and female offenders were reported. In one of these three cases, there was no difference between the sexes with regard to the kyukei and the court sentences (C1). In the other two cases, the ratio for male offenders seemed to be higher than for female offenders, although a statistical analysis was not carried out due to small size of the sample (C3, 4).

**Table:** Table 2:**Case Details of Pair Co-Offenders**

Case(age)	Sex(age)	Victim sex/age	Relationship	Offence	Cause of death	Kyukei(years)	Sentence(years)	ratio
Ｃ1	M(24)	F/ 3y	Non- biological	Hit on the stomach	Peritonitis from duodenal perforation	10	9	0.9
	F(22)		Biological			10	9	0.9
Ｃ2	M(38)	M/ 4y	Non- biological	immersed in cold water	Drowning			
F(25)		Biological				10	7	0.7
Ｃ3	M(25)	M/ 4y	Non- biological	punch and kick against his head	Head injury	10	6	0.6
	F(27)		Biological			8	4	0.5
Ｃ4	M(40)	M/ 16y	Biological	beat, tied, stripped, and then splashed with cold water	Hypothermia	12	10	0.8
	F(40)		Biological			10	7	0.7

“M”=Male, “F”=Female, “y”= year old, “-“ = unreported

## Discussion

The offender group comprised nine biological fathers (32%), 11 biological mothers, and eight non-biological fathers (32%). Of the offenders who were biological mother, three of them were involved in their partner’s assault against their children.

The sex distribution of offenders in child abuse cases appears to be different in different countries. For example, during the same year in the US, according to the National Child Abuse and Neglect Data System, females comprised a larger percentage of child abuse perpetrators than males, 56.5 % compared to 42.4 %.^11^On the other hand, data from Statistics Canada (the nation’s national statistical agency) showed that females who had been accused of assault against children comprised a minority group. In all incidents of assault against children in Canada, the percentage of mothers who had been accused as the perpetrator was 13%, other females 5%, and fathers 44%. The rest were intra/extra-familial male offenders.^12^

In the present research, though there was no difference in kyukei between the sexes, and the court sentences and ratio for females were obviously lighter than for males. As in our previous research, conducted between 1999 and 2007,^7^there was a tendency for the courts to hand down lenient sentences when compared with the kyukei for female offenders. The annual criminal statistics seem to suggest that men receive relatively more severe sentences than women in Japan. For example, the national statistics in 2005 ^13^showed that for those who were convicted of murder, 32.9% of women were given a suspended sentence of imprisonment as compared with 15.7% of men. Conversely, 35.2% of men received prison sentences of over ten years as compared with 15.7% of women.

Farrington and Morris 14 investigated whether the sex of the defendant was related to the severity of sentencing and found that women were sentenced more leniently. However, their research focused on offenders convicted for shoplifting, a petty crime that is the most common offence committed by women, and they conceded that the sex difference disappears after one allows for the fact that women commit less serious offences and are less likely to be previously convicted. In our sample of offenders, even if we made allowance for the existence or non-existence of a criminal record, considering the seriousness of the act of abuse and consequent death of the child, it can be said that the leniency of the court sentences the female offenders was remarkable.

At the same time, the lenient sentence for mothers who killed their children is not peculiar to Japan. d’Orbán^15^reported on the sentences for 89 women charged with the murder or attempted murder of their children during a 6-year period (1970－75) in England and Wales. Of all subjects, 50% were ordered to be hospitalized, 27% were given suspended sentences, and apart from the 2 mandatory life sentences for murder, only 9 other women received a penal disposal. The prison sentences ranged from 18 months to 3 years, with a mean of 26 months of very short term. Of the mothers who had murdered their children, no woman was found guilty of murder, 56% were ordered to be hospitalized, and 65% were not sentenced to prison. These results are is in marked contrast to the sentences received by 29 fathers who killed their children (for 21 fathers, i. e. 72%, the announced imprisonment was not less than 3years; this included 6 life imprisonments).^16^Putkonen et al.^17^analyzed 44 Finnish women who were suspected of neonaticides between the years 1980 and found that only 12 of them were eventually prosecuted. Of these 12, ten offenders (83%) received a sentence of conditional (suspended) imprisonment.

 In more recent research in France, however, of 17 mothers who had killed their own children, 5 were treated with sympathy, 2 were ordered to be hospitalized, 3 were given probation, and the others were imprisoned for not less than 10 years.^18^According to Taguchi,^19^of the 96 maternal filicide cases in Japan, 33.3% were imprisoned (a mean term of imprisonment was 3.4 years, ranging from 0 to 12 years), 63.5% were given a Suspension of Execution of the Sentence, and 3.1% were acquitted due to insanity.

It should be noted that the only instance of a suspended sentence in our study (F5) was that of a 27-year-old mother who had delivered twins and was troubled about providing care for her underdeveloped daughter. Her husband and parents did not support her in raising the child. She had lifted her four-month-old daughter and thrown her onto the bed; however, the court declares that since she had not performed abusive acts on a daily basis, her action was merely an impulsive assault. The judge further justified this sentence by stating that it would have been too harsh to charge the mother with exclusive responsibility for the assault. As described by Pearson,^20^the trend towards lenient sentences for mothers may be due to the popular belief that men are partly responsible for any misconduct committed by their wives.

As of March 2010, there are only two cases that deal with fatal child abuse in the saiban-in system. In the first case, the biological father (age 34 yrs) and mother (age 33 yrs) tied the hands and feet of their four-year-old son with a rope, put a gag in his mouth, and threw him into a closet “in order to prevent him from making a noise” while they were at work. The boy was left unattended for eight hours and eventually died from a heart stroke. The father was sentenced to 9.5 years, while the kyukei was 10 years in jail (ratio: 0.95), and the mother was sentenced to 6 years, while the kyukei was 8 years (ratio: 0.75).

In a second case, the biological father (age 35 yrs) and mother (age yrs35) confined their two-year-old son to a garbage can as punishment for snitching food. The boy died due to suffocation. The father was sentenced to 11 years, while the kyukei was 12 years (ratio: 0.92), and the mother was sentenced to 7 years, while the kyukei was 10 years (ratio: 0.7).

It was observed that a female offender who had depended on a male (principal) offender was likely to receive a more lenient sentence than the male, both in the saiban-in system as well as in the conventional system.

To consider the recent media reports of fatal abuse cases like the Heki-chan case (referred above), abusive act to harmless children will presumably be also found to despicable crime by lay judge.

## Conclusion

Japanese media indentified a trend in Japanese criminal justice system in recent years towards increased leniency in the sentencing of individuals convicted for domestic homicide cases as compared to those involved in instance of public homicide, as well as more stringent penalties for child abuse cases. The present research, however, found no significant alteration in the sentencing of a fatal child abuse cases before and after the preparation period of the saiban-in system.

Following this research, we anticipate that the saiban-in court’s dealings with fatal child abuse cases will increase, and we will continue to observe the sentencing trends for such cases.
